# A P2P Framework for Developing Bioinformatics Applications in Dynamic Cloud Environments

**DOI:** 10.1155/2013/361327

**Published:** 2013-05-09

**Authors:** Chun-Hung Richard Lin, Chun-Hao Wen, Ying-Chih Lin, Kuang-Yuan Tung, Rung-Wei Lin, Chun-Yuan Lin

**Affiliations:** ^1^Department of Computer Science and Engineering, National Sun Yat-sen University, No. 70 Lien-hai Road, Kaohsiung City 80424, Taiwan; ^2^Department of Information Technology, Meiho University, No. 23 Pingkuang Road, Neipu, Pingtung 91202, Taiwan; ^3^Department of Applied Mathematics, Feng Chia University, No. 100 Wenhwa Road, Seatwen, Taichung City 40724, Taiwan; ^4^Department of Computer Science and Information Engineering, Chang Gung University, No. 259 Sanmin Road, Guishan Township, Taoyuan 33302, Taiwan

## Abstract

Bioinformatics is advanced from in-house computing infrastructure to cloud computing for tackling the vast quantity of biological data. This advance enables large number of collaborative researches to share their works around the world. In view of that, retrieving biological data over the internet becomes more and more difficult because of the explosive growth and frequent changes. Various efforts have been made to address the problems of data discovery and delivery in the cloud framework, but most of them suffer the hindrance by a MapReduce master server to track all available data. In this paper, we propose an alternative approach, called PRKad, which exploits a *Peer-to-Peer* (P2P) model to achieve efficient data discovery and delivery. PRKad is a Kademlia-based implementation with *Round-Trip-Time* (RTT) as the associated key, and it locates data according to *Distributed Hash Table* (DHT) and XOR metric. The simulation results exhibit that our PRKad has the low link latency to retrieve data. As an interdisciplinary application of P2P computing for bioinformatics, PRKad also provides good scalability for servicing a greater number of users in dynamic cloud environments.

## 1. Introduction

Today new technologies in genomics/proteomics generate biological data with an exponential growth. Current *Next Generation Sequencing* (NGS) technologies can produce gigabase-scales of DNA and RNA sequencing data within a day at a reasonable cost [1–3]. Cloud computing has been regarded as a key approach for processing such a planet-size data, and hence, many bioinformatics applications have been migrated to the cloud environments [4–7]. Bioinformatics clouds are heavily dependent on data, as data are fundamentally crucial for receiving biological insights. The analyses are commonly based on the extensive and repeated use of comparative parallel process via *Data-as-a-Service* (DaaS) on the web [8–10], most notably in the gene expression analysis. The data are likely to be updated constantly. The sources and users of the data would be connected by various devices over the internet. The effectiveness for locating the deluged data in cloud computing is often overlooked, but it is a key problem. From the aspect of retrieving the up-to-date data with less complexity and delay, we settled the existing problems in data discovery. Along these lines, the high computing ability of P2P framework is adopted as a dynamic cloud infrastructure to resolve the challenge caused by massive datasets [11–13].

Bioinformatics usually requires the collection, organization, and analysis of large amounts of biological data through computers and storage units connected by networks. Many integrative projects attempt to improve the accessibility of biological data and analysis result to end users. P2P computing makes considerable services efficient in a large-scale network, such as file sharing, content distribution, and application-layer multicasting application. In view of this, Montgomery et al. proposed a P2P platform for analysis integration so as to improve the accessibility of algorithms and potentially provide bioinformaticians with unprecedented computing resources [14]. Quan et al. described a method where one can maintain the precision in experimental process within a P2P architecture and presented how this can support experiments [15]. There are two classes, structured and unstructured, of P2P overlay networks, where the former maintains a network topology, and the latter does not. Structured P2P overlay network is often constructed based on *Distributed Hash Table* (DHT), in which the location information of data is placed at peers with identifiers corresponding to the unique key of data. There are many DHT-based P2P systems, such as CAN [16], Chord [17], Chord2 [18], Pastry [19], Tapestry [20], and Kademlia [21]. Lua et al. conducted an extensive survey and comparison of both structured and unstructured P2P overlay network [22].

DHT-based systems have several properties of decentralization, scalability, and fault tolerance. Decentralization means that the network nodes collectively build the system without any central mechanism, scalability represents that the system should work for a great number of nodes with little degradation in performance, and fault tolerance implies the reliability under peers continuously joining, leaving, and failing. For above properties, the nodes of DHT-based P2P systems should communicate with other nodes. Kademlia [21] is one of DHT-based P2P decentralized overlay networks whose identified key is obtained from the SHA-1 hash, and it constructs an XOR-based metric topology to simplify the internal operations. The Kad network implements the Kademlia protocol, while eMule and MLDonkey are two famous P2P file sharing applications supporting the Kad network implementation [23].

Kademlia network follows the XOR metric for distance evaluation, resulting in no delay consideration in the physical network for searching. In other words, the optimal search result given by Kademlia could provide poor link latency. Therefore, we add a mechanism into Kademlia to reflect the link condition of the physical network. Our goal is to select the nodes with the better link latency in cloud environment from all candidates found in the search process of Kademlia. Moreover, our method keeps Kademlia features preserving so as to use its sophisticated operations.

This paper is organized as follows.  Section 2 introduces Kademlia architecture and protocol, together with its problem on poor transmission. Related works are also addressed in this section. Subsequently, we conduct extensive simulations in Section 3, and finally, Section 4 draws our conclusion.

## 2. Materials and Methods

### 2.1. Kademlia

Kademlia is based on the pair of (key, value), and use XOR-metric to be the distance evaluation between two nodes. Both the NodeID and data key are binary sequence of 160-bit length obtained from SHA-1 hash, while the (key, value) pair comes from the physical location of data. The distance is computed by the logical operator XOR of two IDs or between ID and key. Nodes are classified according to XOR metrics of themselves, and there are total 160 buckets due to the NodeID length. A bucket stores the nodes whose XOR metrics are between 2^*i*^ and 2^*i*+1^, 0 ≤ *i* < 160. Because of the upper bound of bucket size *k*, it is also named as *k-bucket* (see Table 1).

The stored nodes in bucket are sorted as the ascending order according to their update time. At the beginning, a bucket is enough to store nodes and will be split once the number of nodes is over *k*, where nodes are classified by their XOR metrics. The default option is to only split the right bucket on the Kademlia tree. If the size of left bucket has achieved the upper bound *k*, Kademlia checks whether a node disappears or not by using the RPC call. For no response from the node, Kademlia removes it followed by adding a new node into the tail; otherwise, it drops the new coming node. That is, *k*-bucket mechanism prefers to keep the nodes with longer online times. Steiner et al. suggested that the longer the online time of a node is, the greater the probability it will be online continuously [24]. In addition, more nodes with long staying time preserved in the Kademlia structure help to make the topology and performance stable.

### 2.2. RPCs and Operations

Kademlia has four RPCs of RTT, STORE, FIND_NODE, and FIND_VALUE. RTT is to check if a node is online, while STORE asks a node to store a pair of (key, value). FIND_NODE retrieves at most *k* nodes nearest the target node with the key. It works by sending a target key of size 160 bits and asking receivers to reply *k* nodes nearest the target node having the key with the form of (IP, ID, Port). Once receiving the response, it repeats the procedure to visit the new retrieved nodes until no new nodes are known. Finally, the mechanism of FIND_VALUE is similar to FIND_NODE but returns the target value with the specified key.

Moreover, Kademlia uses the three operations, Lookup, Publish, and Search. Lookup is based on the FIND_NODE and repeats to send request messages simultaneously to all new nodes it knows. Once a reply message returns some feasible nodes, it immediately sends request messages to these new nodes. The procedure continues until no new node is available. At most *k* nodes nearest the target node with the key are returned in the end. As to Publish, it computes the key as the target and uses Lookup to retrieve *k* nodes nearest the key. Subsequently, it sends request messages to these *k* nodes for asking them to store the information of (key, value). Furthermore, Search operation starts the search process from the list of itself and then uses FIND_VALUE if no target is found. Compared with Lookup, Search terminates its procedure once the value corresponding to the target key is discovered. Eventually, Search operation updates the (key, value) information in the closest node to the node with target key if the closest one contains no or aged information about the specified key.

### 2.3. Node Join/Leave

To join the Kademlia network, the node *x* knows at least a node *y* that has been in the network and starts Lookup targeting to ID. During the Lookup process, *x* can update the bucket content of itself and other nodes. Besides, Kademlia does not regard the node leave as an emergency event. Instead, it removes the offline node only when a new node asks to enter a full *k*-bucket.

### 2.4. Problem

Kademlia employs XOR metric to construct the overlay network without taking the transmission of physical network into account. As a result, the target nodes being the optimal search result returned by Kademlia could have poor performance in coming transmissions. In this paper, we moderate the gap between physical and overlay network of Kademlia by adding a transmission evaluation into Kademlia with preserving its features.

### 2.5. Related Works

Some works have addressed the problem and present their solutions to consider physical network with a direct/indirect way in constructing the overlay network. The direct method is to adjust the ID of each node [25, 26], whereas the indirect way is to add more parameters as filters when performing operations [27, 28]. Yamato et al. used a Locator/ID separation approach with the hierarchical Kademlia for new generation networks, and mobile nodes could retrieve specific data without explicit mapping [25]. The design transformed the device identity from its location into two different namespaces, which are NodeID and Routing Locators (RLOCs). The implementation of hierarchical Kademlia eliminated the need of name servers. However, if a super node leaves, its subsidiary nodes would be forced to disconnect from the network. The reformation of the hierarchy would require considerable time.

Sioutas et al. randomly chose a node to store an index table of the level clustered nodes, and these nodes were assigned an ordered NodeID by a given autonomous ranging factor [26]. In this way, the data searching process with peer churn would only require *O*(log⁡·log⁡*n*) hops for *n* nodes. The approach enabled range query process on large-scale, typically distributed infrastructures, such as clouds of thousands of nodes at shared datacenters. In such distributed environments, range query is the key for managing the distributed data and for monitoring the infrastructure's resources. Through this method the time of network restructuring reduced, but for two nodes nearest to each other being in different clusters, they would take longer time to lookup node. Also, the implementation of autonomous range tree in decentralized architecture is still complex.

 Lai and Yu used the vector of different attributes as key parameters to reserve resources [27]. The resources with the same vector are aggregated to form the attribute groups. These attribute groups connect each other to form a hybrid overlay for decentralized data sharing. The hybrid overlay is a semistructured multistar overlay, which combines structured and unstructured P2P networks to support complex data queries. The multistar overlay connects to the attribute groups to offer range queries and reduced the number of routing hops by migrating requests. Since the focus is the load balancing, the defect of this method suffered excessive delay when transferring redundant connections to other low loading neighbors, especially when the attribute group has never received the same data query before.

In [28], a geographical overlay system was built for geodistance based resource lookup and data delivery. The system managed the overlay network and end system node multicast by utility-driven routing, which involves consideration of the best combine geodistance and link latency (i.e., round trip time). Each node maintained information about *O*(log⁡*n*) other nodes in its list of peer node, and a node must be described with a tuple of five attributes, which are two unique identifiers of geographical location, a region marker, and IP address with port as well as the amount of resource of that node. Regardless of the claim for producing the near-optimal routing decision, the overhead were likely to be excessive, and the scalability of this approach might be an issue.

In this paper, we use the RTT value as the Lookup filter and develop PRKad so as to return targeting nodes with smaller RTT values. A smaller RTT value implies a shorter transmission time, which can reduce the cost of later network transmissions. Besides, PRKad has no structure of the super node and also can avoid selecting farther nodes.

### 2.6. PRKad

**Figure 1:**
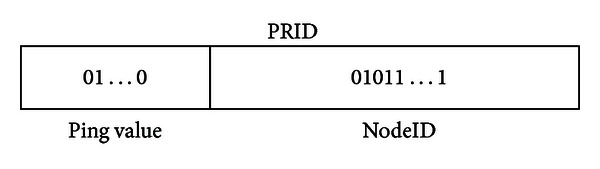
PRID representation.

Similar to Kademlia, NodeID and key in PRKad are binary sequences with length 160 bits, and XOR operation is also used for the distance evaluation. Apart from XOR metric, PRKad utilizes the RTT value to somewhat show how far the physical distance. The RTT value is represented by a binary number of fixed length and put in the prefix of NodeID. We call it PRID shown in Figure 1. PRID information is exchanged during the node communication, with which nodes can know both the physical and XOR distances to each other.

**Figure 2:**
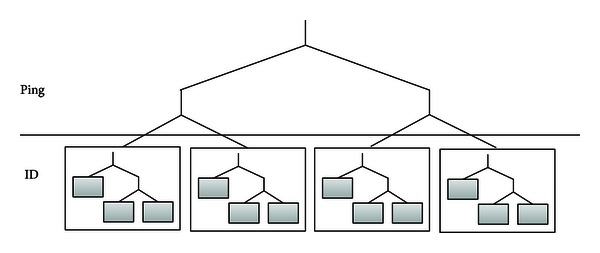
PR tree.

Based on PRID, PRKad constructs a binary tree, named *PR tree*, with a Kademlia tree in its leaf node (Figure 2). When a node enters the PR tree, its RTT value is corresponding to a specific leaf node on PR tree, and next, NodeID is referred to a particular bucket.

**Figure 3:**
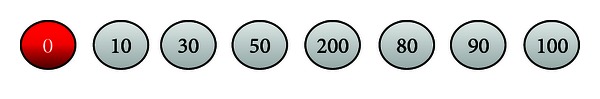
Example of eight nodes with their RTT values.

Suppose that there are eight nodes.  Figure 3 illustrates the RTT values corresponding to the red node whose RTT value is 0. The upper bound of the size of leaf node on PR tree is set to 3, and then, we show the process for other 7 nodes entering the PR tree.

**Figure 4:**
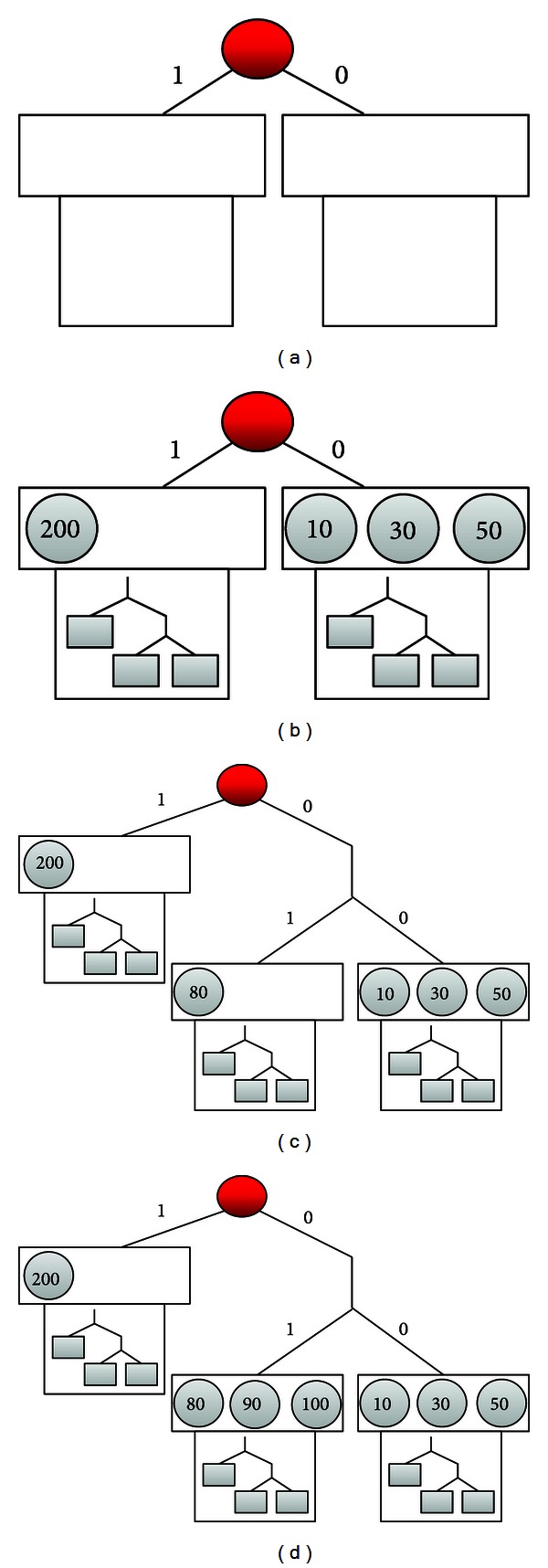
A construction example of PR tree.

At the beginning, PR tree is divided into two PR subtrees by default, illustrated by Figure 4(a), so that we can avoid putting all nodes in a leaf node of PR tree. After four nodes with RTT values of 10, 30, 50, and 200 entering the PR tree, Figure 4(b) shows the result with the tree height 1. Since nodes are classified by the first bit of their RTT values, the right PR subtree contains nodes 10, 30, and 50, and the left subtree has the node 200. Next, the two leaf nodes of PR tree have their respective Kademlia trees according to the NodeID.

When adding the node with RTT value 80, we know that it should be put on the right PR subtree according to the same rule; however, the right leaf node requires to be split due to the upper limit being 3.  Figure 4(c) illustrates the PR tree with five nodes, where the tree height is 2 and nodes are classified with their first 2 bits of RTT values. In other words, the nodes 10, 30, and 50 are on the same Kademlia tree, whereas the node 80 is alone. Finally, Figure 4(d) presents the result once the nodes 90 and 100 are entering the PR tree.

On the other hand, PRKad utilizes five RPCs of BOOT, RTT, STORE, FIND_NODE, and FIND_VALUE, where the usages of the last four RPCs are similar to Kademlia. A node *x* uses Bootstrap operation based on BOOT to enter the network, which works as the following procedures.

(1) *x* picks a node *y* existed in the network and acquires the RTT value with *y* to form the PRID. Next is to send out bootstrap request message with information (IP, ID, and Port).
(2) After receiving the bootstrap request message, *y* extracts PRID from the message and adds the new coming node *x* into *y*'s PR tree. Subsequently, *y* sends response message back with its PRID inside.
(3) For receiving the bootstrap response message from *y*, *x* adds *y* into its PR tree by using *y*'s PRID.
(4) *x* performs Lookup operation to know other nodes in the network.

Here, we set two positive integers *d* and *p*_*t*_ for indicating the thresholds of XOR metric and RTT value, respectively. Lookup operation in PRKad is based on the FIND_NODE with the following procedures.

1. Select *k* nodes at random from the right PR subtree, where *k* is the general bucket size. The *k* nodes comprise a group X, and Lookup operation starts with these nodes (the reason why we exploit *k* nodes here is to increase the probability of selecting nodes with smaller XOR metric at the initial stage. Since nodes have different RTT values, the nodes in the same bucket of Kademlia could be put into different locations of PR tree. Randomly selecting *k* nodes could help to pick appropriate nodes at the beginning).
2. Select *α* nodes from X closest to the target and send Lookup request messages to them.
3. Remove nodes without responses from X and send Lookup request messages to other nodes in X closest to the target.
4. After receiving Look response messages, that is, node list, sort the nodes with their XOR metrics and compute their RTT values.
5. The nodes whose XOR metric and RTT value are less than *d* and *p*_*t*_, respectively, are stored into the list L, implying the success range of Lookup; otherwise, they are added into X.
6. Repeat step (2) until no new node is available or the size of L reaches the upper limit *k*.

The Publish operation of PRKad is identical to that of Kademlia. If we keep the Lookup method for PR tree, then the obtaining nodes are near in cloud environment, leading to the difficulty to distribute file information over the network. As a result, PRKad employs the same publish mechanism in Kademlia to spread information around.

The goal of search operation in PRKad is to find out *k* nodes closest to the target key. It starts to search the list L, and if no target is found, it uses the Lookup operation. However, there are a few differences compared with the above Lookup procedures, shown in the following.

1. Send the search request once a node is added into the list L.
2. Stop if receiving the value corresponding to the target key.
3. For no responses from nodes, we continuously look up to the nodes beyond list L, inspect the *d* and *p*_*t*_ of new coming nodes, and send the search request to the valid node.
4. A node receiving the search request searches the target key in the list L and replies target information if found; otherwise, it returns the node list of particular bucket according to PRID.

Since the Publish operation in PRKad is identical to that in Kademlia, all nodes having data could be put on the left PR subtree, resulting in the poor performance of Lookup operation when starting search from the right PR subtree. Therefore, once there is nothing found within the range of metric *d*, we can pick nodes from the left PR tree immediately to reduce the searching time. Besides, the positive integer *p*_*t*_ helps to filter nodes with distance less than *d* but having a large RTT value.

## 3. Results and Discussion

### 3.1. RTT/Latency

In this paper, the RTT value is an important parameter. In the following experiments, we utilize the formula of PeerfactSim [24] to simulate the RTT value. The formula comes from [29] and is used to compute the latency of two nodes lay on a 2-dimensional plane in PeerfactSim. Given two nodes *s* and *r*, the formula is as follows:

(1) $$\begin{array}{c} \text{Latency}\left(s,r\right)=f\cdot \left(\text{df}\left(r\right)+\frac{\mathrm{dist}\left(s,r\right)}{v}\right), \end{array}$$

where *f* a random number out of (0.1, 0.2,…, 1.0) represents the retransmission probability due to several reasons, for example, congestion, packet loss, and so forth. df(*r*) is the processing time at node *r*, and its value is between 0 and 31 ms depending on different systems. The shortest Euclidean distance between *s* and *r* on the plane is represented by dist⁡(*s*, *r*), and the absolute term *v* is the signal propagation speed of default value 100,000 km/s in PeerfactSim. Therefore, the quotient of dist⁡(*s*, *r*)/*v* denotes the propagation delay of a signal.

We follow the same configurations with [30] so as to make the formula more reliable. The simulated plane is also fixed as 40,000 km × 40,000 km. Other distinctive parameters and values for experiments of Kademlia and PRKad are listed as follows.

- *k* = 20 is the upper bound of bucket size.
- *α* = 3 is the number of selected nodes at a time in step (2) of Lookup operation.
- RTT_NODE_LIMIT = 100 is the maximum number of stored nodes for a node using the RTT call.

### 3.2. Simulation Flow

**Figure 5:**
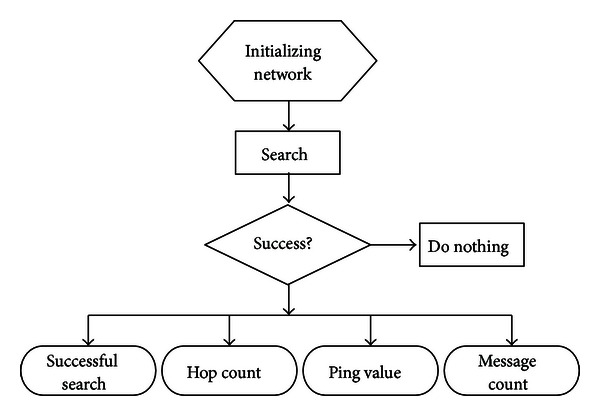
Simulation flow.

Since the Search operation is the most frequently used and contains the Lookup operation in its procedure, our simulations focus on the Search performance.  Figure 5 shows the simulation flow.

*Initializing Network*. We randomly distributed nodes on the simulated plane and then perform the Boopstrap procedure.

*Search*. Each node executes Search operation with ID as the target key. A simulation is complete when all nodes finish their search processes.

*Success?*. We count the following four experimental statistics after the successful searches of nodes. Besides, the node with the smallest XOR metric is the closest node to the host, and for convenience, we call it *XOR closest node* (XCN for short).

(i) *Successful Search*: A search obtaining XCN can be regarded as a successful search, while the number of successful search is increased by 1 if a node discovers its closest node. In the simulations, Kademlia's XCN has the smallest XOR metric to the target key; by contrast, PRKad takes nodes of top *α* (=3) smallest XOR metrics as the XCNs.
(ii) *Hop Count*: The variable calculates the average of the number of hops for each node with a successful search. The hop count of a node is the number of hops reaching the target key and increased by 1 in a pair of request and response:
  (2) Hop  Count  =(∑hop  counts  of  a  node        with  a  successful  search)   ×(Amount  of  nodes  with  successful  searches)−1.
(iii) *RTT Value*: We compute the average of the summation of RTT values to XCNs for each node:
  (3) $$\begin{array}{c} \text{Ping}\;\text{Value}=\frac{\sum \text{ping}\;\text{values}\;\text{of}\;\text{each}\;\text{XCN}}{\text{Amount}\;\text{of}\;\text{nodes}\;\text{with}\;\text{successful}\;\text{searches}}. \end{array}$$
(iv) *Message Count*: We also count the amount of messages used for Lookup requests. Then, the variable calculates the average number of messages used in the successful search for each node:
  (4) Message  Count  =(∑amount  of  messages  for  each  node        with  a  successful  search)   ×(Amount  of  nodes  with  successful  searches)−1.

**Figure 6:**
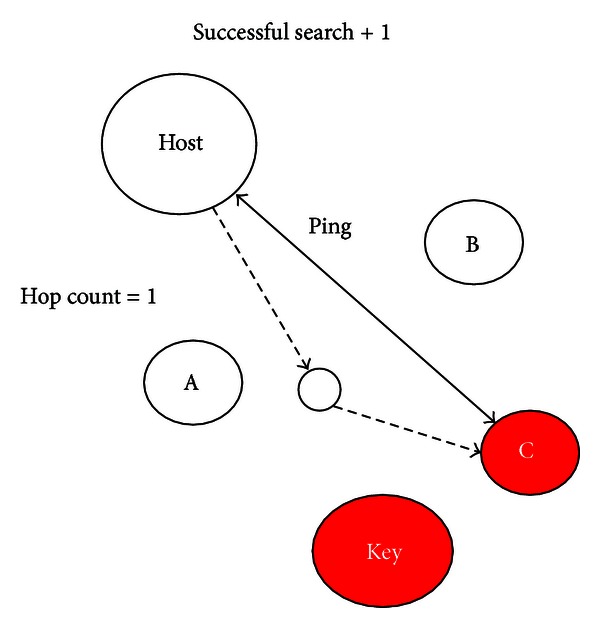
Illustration of a successful search.

Figure 6 illustrates the relations among the above variables we consider. Two closer nodes in Figure 6 indicate the smaller XOR metric they have. Nodes A, B, and C are top 3 XCNs to the target key, and they own identical information. Kademlia uses the node C as its XCN, whereas all three nodes can be the XCNs in PRKad. We set the same range to avoid unfair comparisons in the following experiments. When receiving the XCN, the number of successful search is increased by 1. In Figure 6, the host finds the node C through an intermediate node, and thus, its hop count is equal to 1. Also, the RTT value and number of messages used in the Lookup request are recorded.

### 3.3. Simulation Result

We conduct extensive simulations for comparing PRKad and conventional Kademlia performances under a typical scenario of constantly updated data. We assume each new data update as a new node; thus, we have an increasing number of nodes in the simulations. Each experimental result shown in the following figures is the average of 10 simulations with the same configuration. Particular settings have been introduced in Section 3.1.

**Figure 7:**
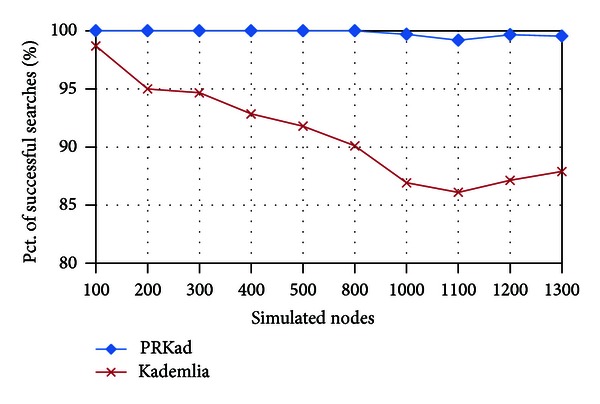
Rate of successful searches.

At first, we show the percentage of successful searches to find out the XCN under different number of nodes. In Figure 7, PRKad's behavior is clearly superior to Kademlia, and the gap is getting large for more number of simulated nodes. The main reason is that if PRKad discovers nothing in the right PR subtree, it continues searching in the left PR subtree. Moreover, PRKad takes *α* (=3) nodes as its XCNs instead of 1 in Kademlia. Two mechanisms raise the percentage of successful searches of PRKad to almost 100 in each simulation.

**Figure 8:**
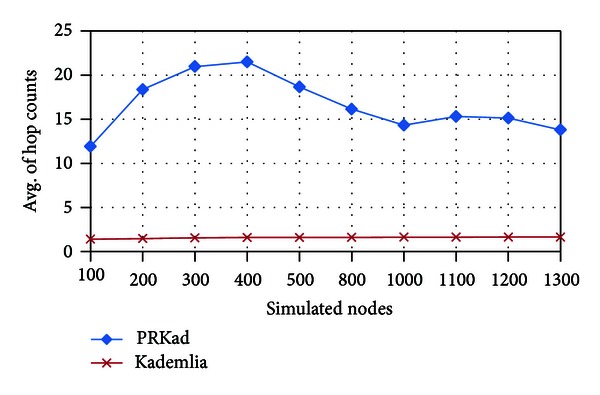
Average of hop counts for successful searches.

Figure 8 exhibits the average number of hop counts in successful searches for different number of nodes. Since we accumulate the number of hop counts for a Lookup, where the right and left PR subtrees may be individually searched with one time, PRKad, as expected, has more hop counts than Kademlia. Furthermore, because small number of nodes on the simulated plane has larger distances between two nodes, the possibility that an XCN appears in the right PR subtree is high. In the situation, PRKad requires more hop counts to search XCNs. As a result, there is a parabola-like opening down for the number of nodes between 200 and 500 in Figure 8.

**Figure 9:**
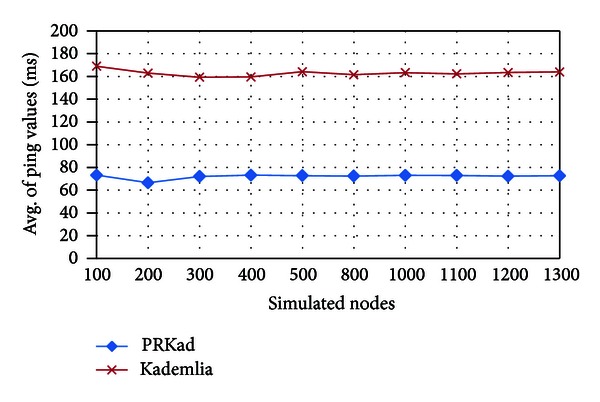
Average of RTT values for nodes with successful searches.

Although more hop counts PRKad has in its searches, it could retrieve XCNs with smaller RTT values. On the contrary, Kademlia has less hop counts but could obtain an XCN having the larger RTT values.  Figure 9 confirms the statement and presents 50% saving for the averages of RTT values in PRkad.

**Figure 10:**
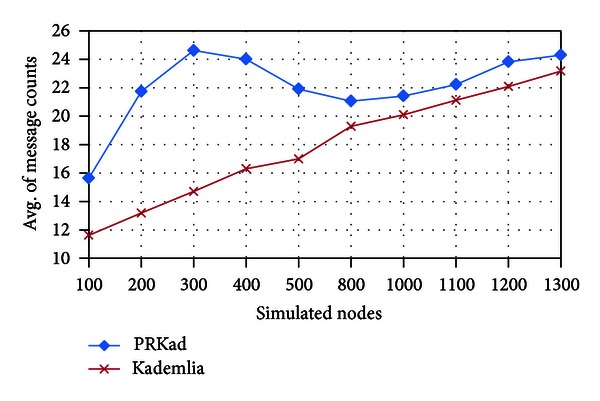
Average of message counts for nodes with successful searches.

Moreover, the average of message counts for nodes with successful searches is an important performance indicator. In Figure 10, the average of message counts in Kademlia is obviously less than that in PRKad, but the gap is becoming small for more simulated nodes. From the above introduction to Kademlia, a node receiving Lookup request replies all nodes in the bucket, and next, the host sends Lookup requests to these nodes. Therefore, the more nodes in a bucket, the larger amount of Lookup requests sent out by the host. Figure 10 presents that the amount of messages used in Kademlia is increasing gradually because more nodes cause more full buckets. In addition, PRKad, unlike Kademlia, does not send requests simultaneously to the nodes given by a Lookup response. Figure 10 also confirms that two parameters *d* and *p*_*t*_ adopted by PRKad can significantly reduce the amount of messages generated by successful searches.

**Figure 11:**
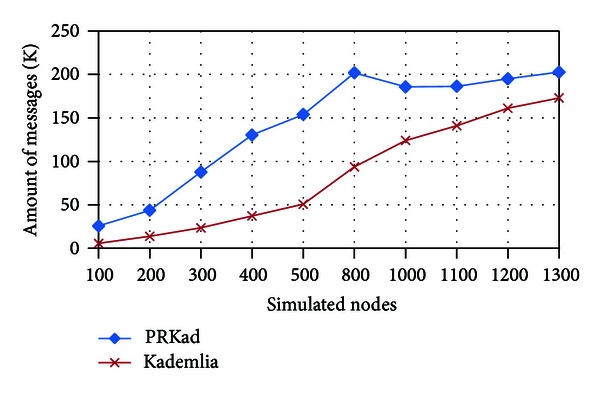
Amount of messages used in simulations.

Figure 11 also shows the amount of messages used in all searches. As expected, the Kademlia curve raises step by step; however, the PRKad curve stops increasing and keeps stable after 800 simulated nodes. The simulation result indicates that the approach of *d* and *p*_*t*_ works well for controlling the amount of used messages.

**Figure 12:**
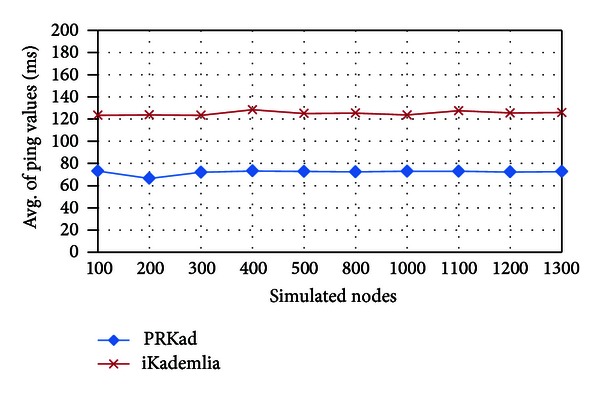
Average of RTT values for nodes with successful searches.

To show that PRKad is indeed better than Kademlia in giving the XCN with a small RTT value, we retrieve the node with the smallest RTT value among all XCN candidates offered by Kademlia. From Figure 12, the improved Kademlia (iKademlia) performs better than original one in terms of giving nodes with small RTT values but is still poorer than PRKad. This implies that during the search process, Kademlia may lose some XCN candidates, which have smaller RTT values.

## 4. Conclusions

Bioinformatics usually requires the collection, organization, and analysis of large amounts of biological data through networks. Therefore, opportunities for applying consistency-checking and data-sharing mechanisms have been found in many areas of bioinformatics. P2P computing makes considerable services efficient in a large-scale network, such as file sharing, content distribution, and application-layer multicasting application. This paper provides a detailed description for the mechanisms of PRkad system and presented a prototype implementation based on the P2P framework and an extensive performance evaluation of the system. By using RTT value as the associate key for searching nodes, PRKad has the small link latency in dynamic cloud scenarios composed of vast simulated nodes. The experimental results show that PRKad outperforms Kademlia, a traditional DHT-based P2P system, in less latency of retrieving data. Though more hop counts PRKad has in its searches, it can retrieve data with smaller RTT values, which assists in transferring large-scale datasets. Moreover, the scalability of PRKad is helpful to serving a greater number of nodes without significantly degrading the performance. PRKad has no job failure even in the presence of very high data updating rates, thus enabling the collaborative applications of data discovery and delivery in bioinformatics.

## Figures and Tables

**Table 1:** *k*-bucket.

Distance	List with *k* = 10
2^0^	■→null
2^1^	■→■→null
2^2^	■→■→■→■→null
⋮	⋮
2^159^	■→■ →■→■→■→■→■→■→■→■→null
